# Early response index: a statistic to discover potential early stage disease biomarkers

**DOI:** 10.1186/s12859-017-1712-y

**Published:** 2017-06-23

**Authors:** Sirajul Salekin, Mehrab Ghanat Bari, Itay Raphael, Thomas G. Forsthuber, Jianqiu (Michelle) Zhang

**Affiliations:** 1 Department of Electrical and Computer Engineering, University of Texas at San Antonio, One UTSA Circle, San Antonio, TX 78207 USA; 2 Department of Biology, University of Texas at San Antonio, One UTSA Circle, San Antonio, TX 78207 USA; 3 Department of Molecular Pharmacology and Experimental Therapeutics, Mayo Clinic, 200 First Street SW, MN, Rochester, 55905 USA

**Keywords:** Disease correlated features, Early stage of disease, Biomarker discovery, Feature selection, Gene/protein expression change, Multiple Sclerosis

## Abstract

**Background:**

Identifying disease correlated features early before large number of molecules are impacted by disease progression with significant abundance change is very advantageous to biologists for developing early disease diagnosis biomarkers. Disease correlated features have relatively low level of abundance change at early stages. Finding them using existing bioinformatic tools in high throughput data is a challenging task since the technology suffers from limited dynamic range and significant noise. Most existing biomarker discovery algorithms can only detect molecules with high abundance changes, frequently missing early disease diagnostic markers.

**Results:**

We present a new statistic called early response index (ERI) to prioritize disease correlated molecules as potential early biomarkers. Instead of classification accuracy, ERI measures the average classification accuracy *improvement* attainable by a feature when it is united with other counterparts for classification. ERI is more sensitive to abundance changes than other ranking statistics. We have shown that ERI significantly outperforms SAM and Localfdr in detecting early responding molecules in a proteomics study of a mouse model of multiple sclerosis. Importantly, ERI was able to detect many disease relevant proteins before those algorithms detect them at a later time point.

**Conclusions:**

ERI method is more sensitive for significant feature detection during early stage of disease development. It potentially has a higher specificity for biomarker discovery, and can be used to identify critical time frame for disease intervention.

**Electronic supplementary material:**

The online version of this article (doi:10.1186/s12859-017-1712-y) contains supplementary material, which is available to authorized users.

## Background

**Fig. 1:**
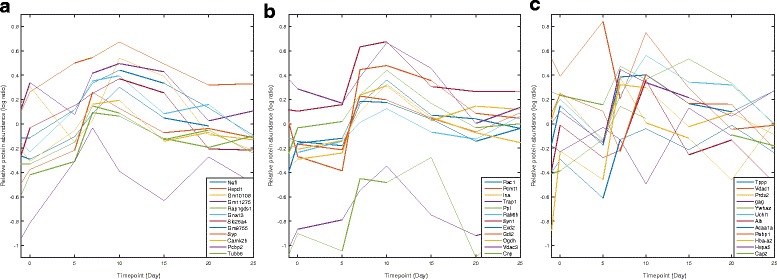
Protein expression time profile across different days for (**a**) proteins identified by ERI at day 5; (**b**) the significant proteins identified by SAM and Localfdr at day 10 but not significant according to ERI at day 5; and (**c**) significant proteins identified by SAM and Localfdr at day 5

Identifying disease correlated molecules at early stages, before the disease process induces high abundance changes in large number of molecules, is a challenging but important problem. The identification will not only lead to the discovery of early diagnostic biomarkers, intervening the disease early for high risk individuals will also be viable. However, before the disease process induces significant changes, most disease correlated biomarkers only have small abundance changes. Several contemporary data mining algorithms are reviewed in [1–3] which include multivariate analysis [4], CART analysis [5], voting panel approach [6], artificial neural network [7], genetic algorithm [8], Significance Analysis of Microarrays (SAM) [9], KTSP [10], and most recently BBPC [11]. However, few progresses has been made towards overcoming the challenge of early diagnostic biomarker discovery. Most feature selection algorithms are designed to boost classification accuracy that favor the detection of features (proteins or genes) with high abundance changes (See Fig. 1(c) for example). Typically, several hundred of candidate biomarkers can be identified at the onset clinical stage with significant abundance changes, and in order to discover a true biomarker for early disease diagnosis, each of them has to be tested using expensive low throughput methods like ELISA to confirm their early involvement [12]. To overcome this problem, gene set analysis based methods [13] has been proposed. However, gene set analysis can only be applied to features with previously known functions, and it does not return individual features that can be tested routinely in low throughput methods. Consequently, there is an urgent need in developing bioinformatic tools for early diagnostic markers detection based on high throughput data.

We address this problem by proposing a new statistics called Early Response Index (ERI) that is sensitive to changes of feature (protein/gene) expression at early stages. Instead of focusing on features that are the most differentially expressed, this index quantifies how much classification accuracy improvement a feature can provide when it is combined with other features in a classifier. The rational is that although a potential biomarker may not have a differential expression that can be regarded as significant by existing tools, its ability in improving classification accuracy may have been significant, and can be detected in high throughput data. Features with significant ERIs can be prioritized for testing as early diagnositic biomarkers. The ERI method is fundamentally different from other feature selection methods [14] that also consider the synergy of features. These “synergistic” methods focus on finding subsets of features that maximize classification performance rather than the *average* performance improvement a feature can bring as in ERI. Consequently, they tend to return features with high abundance changes and miss potential biomarkers suitable for early diagnosis.

We applied the developed ERI algorithm to a locally collected proteomics dataset of experimental autoimmune encephalomyelitis (EAE), and the results has been compared to SAM and Localfdr [15] algorithm, which are typical feature selection algorithms with controlled false positive rates (FPR). Although the EAE dataset is a time series data, most existing time series data processing software [16, 17] are not applicable because these algorithms require a controlling time series data, which is absent in the tested dataset. Other multivariate and synergistic feature selection algorithms such as KTSP [10] are not designed to return a comprehensive list of significantly expressed features, and are not included in our comparison study.

EAE is an animal model of autoimmune neuroinflammatory demyelinating disease and the most common pre-clinical model for studying human multiple sclerosis [18] which is a disease in the central nervous system (CNS). The proteomics dataset was generated using M2-proteomics [19, 20], a previously described quantitative mass spectrometry method which utilizes TMT labels [21, 22]. The analyzed dataset was collected using 160 mice at 8 time points [23]. At each time point, 18 to 20 mice were used to obtain the proteomic expression profile of the mice’s brain homogenate. Day 5 and day 7 are considered as the pre-clinical onset stage of the disease (early stage), when no obvious symptoms are observed; overt symptom appear at approximately day 10 [23]. In this work, our goal was to identify significantly differentially expressed proteins at day 5 which is the pre-onset stage of the disease. Day 5 is considered here because a large number of proteins show significant expression changes after this time and hence, it is a critical turning point of disease development.

As expected, existing data mining tools such as SAM and Localfdr tends to detect many significantly expressed proteins at later stages of disease development. For instance, SAM detected 191 proteins with 0% FPR on day 10, however, only 6.81% of these proteins were detected by SAM on day 5 at the same FPR. Similarly, 152 proteins were identified by Localfdr in day 10 out of which merely 4% proteins were determined as significant by the same algorithm on day 5. In contrast, 25.6% of the proteins identified by both SAM and Localfdr on day 10 can be detected by ERI on day 5. In total, ERI detected 73 significantly expressed proteins on day 5 at 0% FPR, while SAM and Localfdr detected only 35 and 18 proteins respectively in an identical experiment. SAM couldn’t identify 50 out of these 73 proteins that were detected by ERI whereas Localfdr failed to detect 59 of those proteins. Similar results were also obtained on day 25. Identification of a large number of non-overlapping proteins by ERI illustrates the efficacy of the proposed method in early biomarker detection.

Testing results also show that detecting biomarkers earlier on day 5 results in a higher specificity of CNS related pathways in a pathway enrichment analysis using DAVID [24]. The specificity is 80% (4 of 5), 20% (1 of 5) and 100% (1 of 1) using the features selected by ERI, SAM and Localfdr on day 5 (Additional file 1). On the other hand, the specificity is 46 and 21.7% using SAM and Localfdr on day 10. This also highlights the need of detecting biomarkers earlier. ERI method doesn’t enrich any pathways on day 10 because it detects only 2 proteins as differentially expressed at that time point. This result is expected because on day 10, a large number of proteins have high expressions which indicate that day 10 is not an early stage anymore in terms of protein expression changes.

To understand if the ERI algorithm is applicable to clinically collected datasets, we further applied ERI on 9 clinically collected gene expression datasets. Since these datasets are not collected from the early stages, ERI didn’t return as many genes as the other two methods under consideration. However, in 2/3 of the datasets, ERI returned significant number of genes, and this shows the wide applicability of ERI.

The much higher sensitivity of ERI at an early stage offers a new statistical tool for identifying features that are involved in the earlier stage of the disease. Currently, the ERI algorithm is applicable to animal model studies in which the disease causing event is known. In the future, when more high quality retrospective longitudinal clinical data will be available, it is expected that ERI will have broad applications in clinical studies.

## Methods

In the process of developing the early response index (ERI) method, the complete work can be categorized into three sections–colletion of EAE dataset and pre-processing of the data, algorithm development and performance evaluation methodology of the developed algorithm.

### EAE dataset collection

The EAE dataset is a large scale proteomic dataset based on 6-plex TMT labeling and Tandem Mass Spectrometry, which has been collected using 160 mice at 8 time points (day -1, 0, 5, 7, 10, 15, 20, and 25). TMT 6-plex allows the simultaneous quantification of six samples in 6 TMT channels in one LC-MS run so that experimental variation can be reduced [25]. At each time point, 18-20 mice were used to obtain the proteomic expression profile of the mice’s brain. Samples from the 18-20 mice are analyzed using 5 runs each day, and within each run of the 6-plex TMT labeling experiment, channel 1 and 6 were reference channels, and the rests were informative channels reserved for measuring protein expression levels of up to 4 mice. The reference channels measured samples pooled from all mice across all days. For details of the experiments, see [23].

### Pre-processing of the EAE dataset

Peptide identification and quantification were performed using Mascot. For details of searching parameters please see [23]. The experiment runs total 40 times generating 5 datasets per day over 8 days. These datasets were arranged such that, the rows represent different peptides and the columns provide various information of the identified peptides including their abundance measurements on all 6 channels.

Additional file 1: Figure S1 shows the workflow involved in preprocessing the EAE experimental data (Additional file 1). To process raw data, we first merged repeatedly identified peptides within each run by adding their abundance values. Then, to minimize the effect of inherent experimental and biological noise and to eliminate the bias due to experimental factors, we divided the abundance measurements of 4 informative channels by the average of 2 reference channels for each identified peptide. As a result, the variance was reduced by 2 folds and the data became free from experimental bias.

After normalizing using the reference channels, we quantile normalized the 4 ratios on the informative channels across all datasets to remove any channel effects, because the channels were randomly assigned for the 20 biological replicates on each day, which should generate identical ratio distributions across the 4 channels.

We then grouped the quantile normalized peptide ratios by their associated proteins. For a particular protein, its abundance level was obtained by taking the median of ratios for all unique peptides of the protein measured in the same channel in each run.

After protein quantification, we unionized all quantified proteins across all 8 time points, and obtained a 734-by-8 cell structure in Matlab, where 734 rows correspond to all of the proteins quantified across all days. The columns correspond to 8 time points. Each cell contains the expression measurement of 20 mice for a particular protein on a certain day.

Due to the selective nature of tandem mass spectrometry, only a fraction of all fragmented peptides has been identified in each run as well as most of the proteins were not quantified in all TMT channels. To address the problem of missing values, we discarded proteins that have less than 12 measurements out of 18-20 total measurements. For proteins with 12 or more measurements, we replaced the missing values with measurements randomly sampled from the 12 or more existing measurements. After this step, 313 proteins remained for day 0 and day 5.

### Description of clinical datasets

To assess if ERI can be applied generally, we downloaded and and applied ERI method on 9 clinically collected datasets. Table 1 provides a summary of the datasets. Six of the datasets have binary classes, while the GSE14333, GSE27854 and CNS datasets are multi-classes. We are focused on studying the two class problem, and in the GSE14333 dataset, patients having colorectal cancer (CRC) of stage I and II tumors are combined as single class representing non-invasive tumors, while patients with stage III tumors, which represent invasive tumors are treated as another class. In the GSE27854 dataset, CRC patients with stages I and II were defined as one class, while stage III and IV patients are combined as other class. In the CNS dataset, the original study was composed of three different sets of samples (Dataset A,B,C) ranging from children with medulloblastomas to adults with malignant gliomas. We analyzed dataset C only, which consists of medulloblastoma survivors and non-survivors.

**Table 1:** Summary of clinical datasets used in this study

Dataset	Genes	Samples class size		No of features		Source
			(ERI)	(SAM)	(Localfdr)	
GSE14333	54675	138/91	0	2	0	[39]
GSE27854	54675	57/58	0	0	0	[40]
CNS	7129	21/39	4	0	2	[41]
Colon Cancer	2000	40/22	3	46	8	[42]
GLI-85	22283	26/59	51	1458	1198	[43]
Lung Cancer	7129	24/62	0	0	0	[44]
Prostate Cancer	10509	50/52	11	946	769	[45]
SMK-CAN-187	19993	90/97	8	289	271	[46]
Breast Cancer	22283	138/71	5	14	2	[47]

### The Algorithm for calculating Early Response Index

Early Response Index (ERI) is calculated in the following process. Suppose a pair of features are *F*_*i*_ and *F*_*j*_, and we use them as features of a Support-Vector-Machine (SVM) classifier [26]. Suppose the classification accuracy rate achievable is *A* *c* *c*(*F*_*i*_,*F*_*j*_) when the SVM classifier is trained using the expression levels of *F*_*i*_ and *F*_*j*_ in a training sample set and evaluated on a testing set in a cross-validation scheme. Suppose *A* *c* *c*(*F*_*i*_) and *A* *c* *c*(*F*_*j*_) represent the classification accuracies if only *F*_*i*_ or *F*_*j*_ are used as the SVM feature. We define the performance improvement due to combining *F*_*i*_ and *F*_*j*_ as the improvement score (IS) of *F*_*i*_ due to *F*_*j*_,

1 $$  IS(F_{i},F_{j})=Acc(F_{i},F_{j})-max\left[Acc(F_{i}),Acc(F_{j})\right],  $$

We calculate this improvement score for all possible combinations of features, and the overall early response index (ERI) of a feature *F*_*i*_ is obtained by taking the average of all improvement score of *F*_*i*_ when it is combined with other features:

2 $$ ERI(F_{i})=\frac{\sum_{j,j \neq i}IS(F_{i},F_{j})}{N-1},  $$

where *N* is the total number of proteins.

We can see that ERI only quantifies the average performance improvement due to a protein feature when it is combined with other features regardless of the maximum accuracy achievable by individual features. It will not favor features that is highly differentially expressed as in other feature selection algorithms.

We selected SVM classifiers for calculating the accuracies because it gives us high sensitivity. We investigated the possibility of replacing SVM with Naive Bayes (NB) [27] and Random Forest (RF) classifier [28]. When using NB, only 13 proteins with significant expressions were detected at 0% FPR on day 5 in the EAE dataset, which is 82% less than that using SVM. Similarly, ERI method identifies only 10 proteins as differentially expressed between day 0 and 5 while using RF instead of SVM as classifier. Moreover, 92% (12 of 13) and 80% (8 of 10) of the proteins detected by NB and RF consecutively were also detected by SVM. Hence, there is little need to calculate ERI again using these classifiers after employing SVM.

### Feature selection process using ERI

**Fig. 2:**
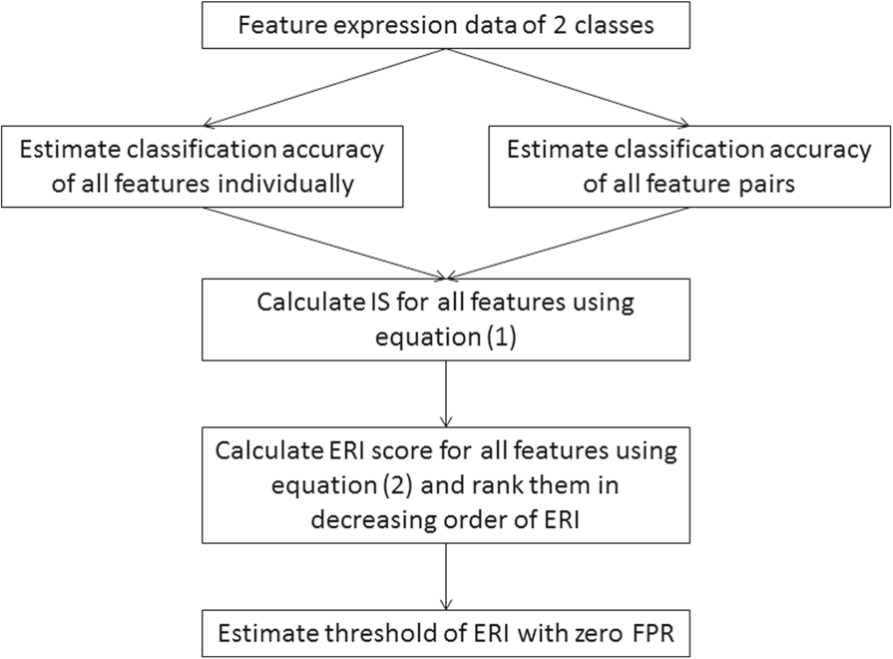
Feature selection flow diagram of ERI algorithm

To apply the ERI as a ranking criteria for feature selection, the processing steps were performed as shown in the flow diagram (Fig. 2).

#### Estimate classification accuracy of individual features

To estimate the classification accuracy of single features (protein or gene), we used a 5-fold cross validation scheme [29] to avoid the problem of overfitting. We trained SVM classifiers using 80% of samples from both classes, and evaluated the performance using the rest 20% of samples. In order to mitigate any effect of selection bias [30], these steps were repeated 5 times by randomly dividing the samples into 5 folds each time. Finally, the average accuracy over the 5 repetitions of 5-folds cross validation was estimated as the classification accuracy achievable (see Additional file 1).

#### Estimate classification accuracy of all pairs of features

**Fig. 3:**
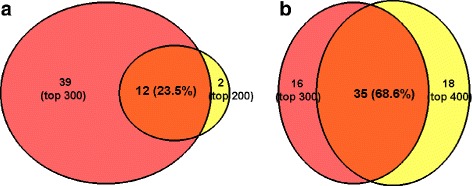
Comparison of overlap of discovered significant proteins between selecting (**a**) 200 and 300 features; (**b**) 300 and 400 features in pre-filtering step

If there is a large number of features in a dataset, estimation of the accuracy of all feature pairs poses a significant challenge on computational complexity. To reduce the complexity, t-test based pre-filtering scheme can be used to reduce the number of features as described in [3]. We found that when we ranked features based on t-test statistics, using more than 300 features does not increase the sensitivity of ERI feature selection (Fig. 3). In the EAE dataset, since there were only 313 proteins measured on both day 0 and day 5, all of them were used. In other datasets, the top 300 proteins are kept.

After pre-filtering, we considered all possible combinations of pairs of the remaining features, and estimated the accuracy achievable using pairs of features in the same procedure as that for individual features.

#### Calculation of improvement score (IS) and early response index (ERI)

The improvement score (IS) of a feature was calculated according to Eq. 1. To determine the ERI score of the feature, the average of all its IS score was calculated (Eq. 2). This process was repeated for all features. ERI stands for the average performance improvement a feature can bring when it is combined with other features for classification.

### ERI cutoff threshold estimation based on FPR.

To determine the cutoff threshold on ERI for selecting statistically significant features at 0% false positive rate (FPR), we employed a permutation scheme as that in SAM, in which the class labels of samples were randomly permutated. Subsequently, the ERI scores were calculated for all features using the procedure as described in the flow chart in Fig. 2. Cutoff threshold was estimated at 0% FPR because we intended to show the usefulness of our algorithm under the most stringent criteria. Since the class labels were randomly permuted, it is expected that none of the features would have a high ERI score. The procedure was repeated 10 times, and the overall maximum ERI score achieved in this repetitive experiment was selected as cutoff threshold. A higher number of permutation trials guarantees more robust threshold estimation. However, the time complexity increases with the increasing number of permutation trials.

Selection of maximum ERI score as cutoff threshold ensures the 0% FPR in true dataset. For example, in our test between day 0 and 5 in the EAE dataset, the maximum ERI score achieved by any protein out of the 10 random permutation trials was 0.0258. Hence, when we set the ERI cutoff threshold as 0.026, it ensures a zero FPR for proteins with ERIs above 0.026. The ERI threshold was calculated independently for each of the tested dataset.

### Early response feature selection algorithm evaluation

The goal of the ERI algorithm is to prioritize and confirm the early involvement of disease correlated features as potential early diagnosis biomarkers. Thus, the number of statistically significant features detected at an early stage of diseases can be used to evaluate the performance of various algorithms.

In this study, we compared the number of significant proteins detected by ERI, SAM and Localfdr at different days using day 0 as reference. SAM had been chosen as one of the comparable algorithms because this method ranks features with a significance score at controlled FPRs. According to [31], in which the authors compared six methods for identifying differentially expressed genes across multiple conditions, SAM is one of the best-performing methods when the sample size is greater than 6 (we have 40+ samples in our tests). SAM has been also used widely for analyzing differentially express genes for various disease conditions until very recently [32, 33]. In addition, Localfdr has been shown to perform competitively in presence of large noise variance [31]. Since, ERI is focused on identifying disease correlated features at early stage when the expression changes in real biomarkers are minimal and vulnerable to be subdued by other noisy features, we also included Localfdr for performance comparison. There are existing feature selection algorithms such as KTSP [10] and MRMR [34] that strive to improve class prediction performance but these algorithms are designed to return a minimal number of features, which makes them inappropriate to be compared with ERI for the purpose of sensitive detection.

Under 0% FPR, we used the R-package of SAM called “samr” [35] to select a list of significant differentially expressed proteins at various time points.

## Results and discussion

### Detecting early responding features

#### Number of significant features identified at day 5 and 25

In the EAE dataset, day 5 is the pre-onset clinical stage when the disease symptoms are not obvious. As our experimental data have shown, hundreds of proteins will have high abundance changes after this time point, and identifying biomarkers that have responded to the disease on day 5 will offer opportunities for disease intervention before a large number of proteins are affected.

**Fig. 4:**
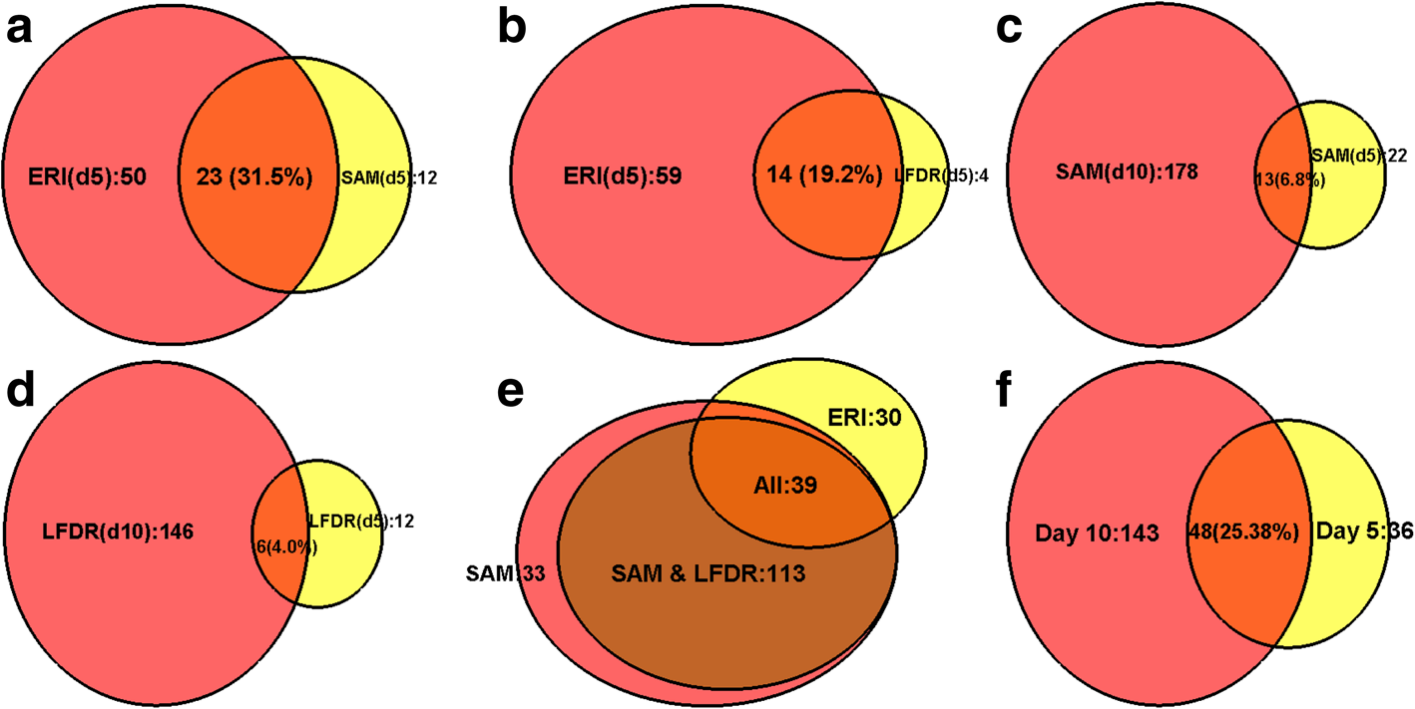
Comparison of overlap of discovered significant proteins (**a**) between ERI and SAM at day 5; (**b**) between ERI and Localfdr at day 5; (**c**) between ERI at day 5 and SAM, Localfdr at day 10; (**d**) between SAM at day 5 and SAM at day 10 (**e**) between Localfdr at day 5 and Localfdr at day 10 and (**f**) between day 5 and day 10 by ERI, SAM and Localfdr combined

We first applied the comparable methods between day 5 and day 0. The ERI algorithm was applied at 0% FPR with a cutoff score of 0.026 which identified 73 proteins as significantly expressed (ERI score ≥0.026). In contrast SAM and Localfdr detected 35 and 18 proteins respectively at 0% FPR. Out of the 73 ERI identified proteins, 50 proteins were not identified by SAM (Fig. 4 a) while Localfdr missed 59 of those proteins detected by ERI (Fig. 4 b). The results between day 0 and day 25 are similar. Day 25 marks the initiation of the remission process which is characterized by clinical attacks (relapses) with diverse neurological dysfunctions, followed by functional recovery (remission) [21]. Though, ERI detected 38 significant proteins at this stage, SAM and Localfdr detected 18 and 7 proteins respectively. Among the 38 ERI proteins, only 26.3 and 5.2% can be detected consecutively by these methods. Interestingly, we also discovered that 50% of the significant proteins identified by ERI during the remission stage were also identified by ERI during the initial stage of the disease.

This shows that ERI is capable of detecting more significant proteins than both competitive methods before critical turning points of disease development.

#### Repeatability of significant feature detection

We investigated how many proteins that were detectable on day 10 could be detected earlier on day 5. While SAM detected 191 significant proteins on day 10, only 13 (6.8%) of the 191 proteins can be detected on day 5 by SAM alone (Fig. 4 c). Similarly, out of the 152 proteins identified by Localfdr as differentially expressed on day 10, only 6 proteins (4%) were detected on day 5 too by the same method (Fig. 4 d). The proteins detected by Localfdr on day 10 completely overlaps with those detected by SAM on the same day (Fig. 4 e) which shows that both the methods perform identically during disease onset and the proteins identified are truly differentially expressed. When we applied ERI, 39 of the 152 proteins (25.6%) that were detected by both methods on day 10, can be detected on day 5 (Fig. 4 e). Combining ERI with SAM and Localfdr together can significantly increase the number of repeatedly identified proteins. Altogether, the three methods identified 193 unique proteins at day 10 and 84 proteins at day 5, out of which 48 are overlapping proteins (Fig. 4 f). We also noticed that 60.27% of the total proteins identified by ERI at day 5 were detected by SAM and Localfdr at day 10. The result is similar when we examine the overlap between other days.

This shows that ERI can verify the involvement of more disease correlated proteins at an earlier stage than SAM and Localfdr. In addition when ERI is combined with those methods, it is possible to identify more features that are responding to the disease process at multiple stages of the disease.

#### Expression profile over time for significant proteins

In Fig. 1(a), we plot the average expression time profile of 12 arbitrarily picked proteins from the 73 proteins identified by ERI on day 5. Only 12 proteins are randomly picked in the expression profile plots because showing more profiles clogs the view. We can see that most of these proteins have shown sustained and monotonically increasing patterns between day 0 and day 10. In contrast, in Fig. 1(b), we plot the expression profiles of 12 randomly selected proteins from the list identified by both SAM and Localfdr on day 10, but were not called as significant by ERI on day 5. Even though these proteins also show the monotonically increasing pattern as in Fig. 1(a), but they have minimal changes between day 0 and day 5. This explains why these proteins were identified by other methods at day 10 but not by ERI at day 5. For comparison, the average expression time profiles of 12 randomly picked proteins from those identified by SAM and Localfdr at day 5 are shown in Fig. 1(c). It can be seen that these proteins generally have little correlation with disease development from day 0 to day 10, and their expression levels cannot track disease development.

**Fig. 5:**
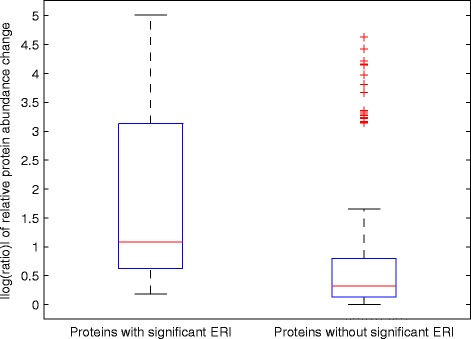
Relative protein abundance change between day 0 and 5 for proteins having significant ERI score and those without significant ERI but picked up by SAM and Localfdr on day 10

A comprehensive comparison of average ratio change of ERI picked proteins (73) and those detected by other algorithms on day 10 but not by ERI (146) on day 5 is shown in the boxplot of Fig. 5. The protein abundance ratios were calculated as the median expression value of proteins across 18-20 samples at day 0 over the the median expression value of these proteins at day 5. The absolute value of all logarithmic protein ratios were used to create the box plot. The box plot shows that ERI identified proteins have higher differential expression levels than those not identified by ERI. Therefore, they should be prioritized as potential early diagnostic biomarkers.

#### Performance of ERI after day 5

It shall be noted that ERI is very sensitive to early changes of protein expression, but not very sensitive to later stage changes. We applied the ERI algorithm between day 0 and all other time points, and the number of significant detection is listed in Table 2. These numbers are compared with that of SAM and Localfdr. We noticed that from day 7 to day 20, SAM detects more proteins than ERI when large number of proteins showed significant abundance changes, whereas ERI detects more on day 5 before significant expression changes occurred in large number of proteins (see Fig. 1 a), which indicates that ERI is more sensitive to the changes in feature expression due to the disease. Similar pattern is evident between ERI and Localfdr as well except day 15 and 20 when the number of detected significant proteins are close to each other. These results imply that ERI method should be applied for early stage biomarker discovery.

**Table 2:** Number of significant proteins identified by three methods across different days of EAE dataset

Method	Day 5	Day 7	Day 10	Day 15	Day 20	Day 25
ERI	73	5	2	13	23	38
SAM	35	106	191	17	27	18
Localfdr	18	219	152	5	21	7

From Table 2, it becomes clear that whether ERI can detect a significant amount of features depends on when the samples are collected. So, the relative number of significant features identified by ERI and other methods can be utilized to infer the time frame of disease development roughly. If SAM and Localfdr detects more than ERI, then it is likely that the disease development has passed its initial stage. ERI and SAM can be applied jointly to detect potential disease biomarkers through the whole course of a disease.

### Biological relevance of identified early stage proteins

To understand the biological relevance of proteins detected by ERI, SAM and Localfdr on day 5, we performed a pathway enrichment analysis through DAVID [24]. It turns out that the 73 proteins identified by ERI on day 5 enriched five KEGG pathways with *p*-value less than 0.05. The pathways are Glycolysis/Gluconeogenesis, gap junction, Parkinson’s, Huntington’s and Amyotrophic Lateral Sclerosis (ALS) diseases, 4 of which are similar to Multiple Sclerosis disease pathways that affect the central nervous system (CNS). In contrast, feeding DAVID separately with proteins detected by SAM and Localfdr on day 5 resulted into only one CNS specific pathways each with *p*-value <0.05 (see Additional file 1). Considering the fact that multiple sclerosis is a CNS disease, it signifies the ERI can return more disease related pathways.

We have also examine the pathways detected by SAM and Localfdr at day 10, and compared with those detected at day 5 using ERI. SAM returned 19 enriched KEGG pathways having *p*-value less than 0.05, and 7 of them were CNS specific. On the other hand, 23 pathways were enriched with *p*-value <0.05 by Localfdr day 10 proteins out of which five were CNS related. By dividing the number of detected CNS pathways by the number of total number of significant pathways, ERI’s specificity in finding CNS related pathways is 80% on day 5, while SAM and Localfdr are only 37 and 21.7% specific on day 10. These results show that performing biomarker discovery at an earlier stage using ERI could potentially increase the specificity of biomarker discovery. The list of detected pathways are provided in Additional file 1.

### Wide applicability of ERI

To assess the applicability of ERI method on human dataset, we have applied the algorithm at 0% FPR on 9 datasets collected from the literature (Table 1). These datasets were collected in clinical settings after the onset of the disease. Thus, they cannot be used to test the efficacy of ERI in discovering early diagnostic biomarkers. However, some of the data are still expected to return significant features as in the case of EAE dataset on day 25.

Since there are thousands of measured genes in these clinical datasets, we used a pre-filtering step to reduce the number of features to 300 based on their t-test *P* values. To test if 300 is an appropriate number, we applied the procedure of calculating ERI scores for the GLI-85 [43] dataset by keeping 200, 300 and 400 genes after the pre-filtering step. We found that when we increase the number of genes from 200 to 300, the number of significantly detected genes by ERI increases significantly as shown in Fig. 3(a). However, when we increased the number of features to 400, the total number of detected features does not increases further (Fig. 3 b). There is also a significant overlap when selecting different number of pre-filtered features. Twelve of the 14 proteins detected when keeping 200 features are found again with 300 features. The overlap between using 300 and 400 features is 68.6%.

The number of detected significant proteins for the 9 datasets by the methods under consideration are listed in Table 1. It can be seen that at least in 2/3 of the datasets, there are a significant number of genes identified by ERI indicating that the method can be applied broadly. For most of the dataset, SAM and Localfdr clearly outperforms the ERI algorithm in terms of the number of significant genes identified. These results are not beyond expectation because in clinical settings, the datasets are usually collected after the onset stage of diseases. When there exists a significant number of ERI detected genes, it is indicative that the disease is progressing to another stage, since ERI only detects early responders to a disease condition.

Notably, ERI identified 4 significant genes for the CNS dataset whereas Localfdr detected 2 significant genes and SAM failed to detect any. The CNS dataset has 60 samples, which includes 39 cases of medulloblastoma survivors and 21 treatment failures. All tumor samples were obtained at the time of initial surgery prior to treatment. Failure of SAM in discovering any significant genes is attributed to the strict false positive rate (0% FPR) that we have applied. This is a more stringent criteria for significance detection than in the original work [41], in which the author discovered a list of 50 markers using a signal-to-noise statistic. Interestingly, B-50 neural phosphoprotein (GAP43), one of the 4 genes identified by ERI method, has been consistently proven to be down-regulated in an independent work by the deficiency of nitric oxide synthase (Nos2) which is indirectly involved in controlling proliferation and differentiation of medulloblastoma developmental process cells [36]. ERI also detected LTC4S, which has also been identified in the list of common cancer signature genes [37]. Another significant gene identified by ERI in this dataset was Endothelial-3 (END3). Though, there is no reference of END3 to be directly involved with medulloblastoma patients but this gene has been reported to be highly produced by glioblastoma stem cells [38], which is a subtype of malignant brain tumors (Glioma) along with medulloblastoma. These results show that ERI can return disease correlated genes even when SAM failed to return any.

## Conclusion

In this paper, a novel statistics, Early Response Index (ERI) is proposed for the detection of disease correlated features for early diagnostic biomarker discovery. The proposed method is shown to have significantly higher sensitivity in biomarker detection compared to SAM and Localfdr before critical turning points of the disease process, after which large number of molecules will be impacted by the disease with significant abundance changes. Furthermore, ERI is sensitive to the time of sample collection. ERI only returns significant number of features before critical turning points when the disease is about to impact a large number of molecules. Consequently, ERI can be used for identifying the best time frame for disease intervention.

Besides increased sensitivity at early stage, ERI has also been shown to have a higher specificity in returning central nervous system (CNS) related pathways when it is used early during the disease process than using SAM at a later stage. This further illustrates the need for using ERI at an earlier stage for more specific and more sensitive biomarker discovery.

In summary, the ERI method has been shown to be very sensitive for significant feature detection before critical turning points of disease development within the scope of Multiple Sclerosis and some clinical cancer dataset used in this study. It potentially has a higher specificity for biomarker discovery, and can be used to identify critical time frame for disease intervention.

## Additional file

Additional file 1 Proteins detected by different methods in different days.xlsx — This file lists all the significant features detected by various methods at different timepoints on EAE dataset. (XLSX 34.1 kb)

## Abbreviations

ALS: Amyotrophic lateral sclerosis
CNS: Central nervous system (CNS)
CRC: Colorectal cancer
EAE: Experimental autoimmune encephalomyelitis
ERI: Early response index
FPR: False positive rate
IS: Improvement score
NB: Naive bayes
RF: Random forest
SAM: Significance analysis of microarrays
SVM: Support vector machine
